# Statistical Frequency-Dependent Analysis of Trial-to-Trial Variability in Single Time Series by Recurrence Plots

**DOI:** 10.3389/fnsys.2015.00184

**Published:** 2016-01-14

**Authors:** Tamara Tošić, Kristin K. Sellers, Flavio Fröhlich, Mariia Fedotenkova, Peter beim Graben, Axel Hutt

**Affiliations:** ^1^Team Neurosys, InriaVillers-lès-Nancy, France; ^2^Loria, Centre National de la Recherche Scientifique, UMR no 7503Villers-lès-Nancy, France; ^3^Université de Lorraine, Loria, UMR no 7503Villers-lès-Nancy, France; ^4^Department of Psychiatry, University of North Carolina at Chapel HillChapel Hill, NC, USA; ^5^Neurobiology Curriculum, University of North Carolina at Chapel HillChapel Hill, NC, USA; ^6^Department of Cell Biology and Physiology, University of North Carolina at Chapel HillChapel Hill, NC, USA; ^7^Department of Biomedical Engineering, University of North Carolina at Chapel HillChapel Hill, NC, USA; ^8^Neuroscience Center, University of North Carolina at Chapel HillChapel Hill, NC, USA; ^9^Department of German Studies and LinguisticsBerlin, Germany; ^10^Bernstein Center for Computational NeuroscienceBerlin, Germany

**Keywords:** trial-to-trial variability, time-frequency analysis, local field potentials, recurrence plot analysis, statistical inference, surrogate data, anesthesia, ferret

## Abstract

For decades, research in neuroscience has supported the hypothesis that brain dynamics exhibits recurrent metastable states connected by transients, which together encode fundamental neural information processing. To understand the system's dynamics it is important to detect such recurrence domains, but it is challenging to extract them from experimental neuroscience datasets due to the large trial-to-trial variability. The proposed methodology extracts recurrent metastable states in univariate time series by transforming datasets into their time-frequency representations and computing recurrence plots based on instantaneous spectral power values in various frequency bands. Additionally, a new statistical inference analysis compares different trial recurrence plots with corresponding surrogates to obtain statistically significant recurrent structures. This combination of methods is validated by applying it to two artificial datasets. In a final study of visually-evoked Local Field Potentials in partially anesthetized ferrets, the methodology is able to reveal recurrence structures of neural responses with trial-to-trial variability. Focusing on different frequency bands, the δ-band activity is much less recurrent than α-band activity. Moreover, α-activity is susceptible to pre-stimuli, while δ-activity is much less sensitive to pre-stimuli. This difference in recurrence structures in different frequency bands indicates diverse underlying information processing steps in the brain.

## 1. Introduction

Investigation of metastable states (MS) and transients of complex dynamical systems has become increasingly important over the last decades. In this context, dynamical systems spend longer time intervals in MSs than in transients between MSs. The large interest in studying such states comes from the belief that a complex temporal behavior of systems may be decomposed into a simple sequence of alternating MSs and transients between them. This reduced description is a model that captures the essential dynamic elements of rather complex underlying dynamics. Applications range from spin glasses (Larralde and Leyvraz, 2005) to molecular configurations (Deuflhard and Weber, 2005) and geoscientific applications (Froyland et al., 2007). In neuroscience, the related concept of sequential metastable attractors has received increasing attention in the last years (Friston, 1997; Oullier and Kelso, 2006; Rabinovich et al., 2008b; Yildiz and Kiebel, 2011; Hudson et al., 2014; Tognoli and Kelso, 2014). Primarily, works are motivated by the experimental observation of signal features showing alternations of dynamical behavior at fast and slow time scales (Hutt and Riedel, 2003; Hutt, 2004; Mazor and Laurent, 2005; Allefeld et al., 2009).

Originally the concept of metastability refers to slow relaxation dynamics in statistical physics (Larralde and Leyvraz, 2005; Tokman et al., 2011). In a much wider sense, this notion is nowadays used for regions in the phase space of a dynamical system with relatively large dwell that are connected by transients (Friston, 1997; Rabinovich et al., 2008b; Tognoli and Kelso, 2014). Paradigmatic examples for those MSs are *almost invariant sets* (Froyland, 2005) and *recurrence domains* (beim Graben and Hutt, 2013), such as saddles connected by heteroclinic trajectories (Rabinovich et al., 2008a) or the “wings” of the Lorenz attractor (Lorenz, 1963). For this attractor in particular, it is attractive itself and has two recurrence domains centered around two unstable foci. Geometrically, these domains are spatially separated and the system's trajectory alternately approaches to and departs from the foci. The system spends much longer time in the vicinity of a focus compared to transient intervals between the two foci. Therefore, one may refer to a Lorenz wing as to a MS: the system remains for a longer time in one partition cell of the phase space before it performs a rapid transition to another partition cell of the phase space. A MS is thus identified with a recurrence domain, while non-recurrent portions of a trajectory can be compared with transients.

In neuroscience, metastability assumed increasing experimental evidence over recent years. Lehmann et al. (1987), Wackermann et al. (1993) observed sequences of metastable electroencephalogram (EEG) topographies, which they called *brain microstates*. Hutt and Riedel (2003), Hutt (2004), beim Graben and Hutt (2015) argued that components of the event-related brain potentials (ERPs) reflecting perceptional and cognitive processes could be identified with metastable brain states. Mazor and Laurent, for instance, reported sequences of metastable states in a reconstructed activation space of the locust's neural odor circuit (Mazor and Laurent, 2005). Allefeld et al. (2009) were able to detect metastable states in epileptic EEG time series through spectral clustering methods, and most recently, Hudson et al. (2014) revealed metastable transition networks in the recovery from anesthesia. Consequently, to understand underlying neural mechanisms much better, it is necessary to develop advanced techniques to detect these recurrence structures in experimental time series.

For the identification of metastability in time series, their characteristic slow time scales must be separated from the fast dynamics of phase space trajectories. The method known as *Perron clustering* (Deuflhard and Weber, 2005), separates the system's phase states into partitions that can approximate Markov chain states (Deuflhard and Weber, 2005; Froyland, 2005; Larralde and Leyvraz, 2005; Gaveau and Schulman, 2006; Allefeld et al., 2009). Applying spectral clustering methods to the resulting transition matrix yields the time scales of the process, while their corresponding (left-)eigenvectors allow the unification of cells into a partition of metastable states (Gaveau and Schulman, 2006; Allefeld et al., 2009). Another approach by Hutt and Riedel (2003) utilizes the slowing-down of the system's trajectory in the vicinity of saddles by means of phase space clustering. Most recently, beim Graben and Hutt suggested to combine recurrence plot techniques and symbolic dynamics in order to partition a system's phase space into its recurrence domains (beim Graben and Hutt, 2013, 2015). The application of the latter method to experimental event-related potentials has identified metastable attractors to so-called ERP-components, known to reflect cognitive processing stages in neural information processing.

Developing novel analysis tools for representation and tracking of non-linear transient patterns faces numerous challenges, such as reducing the signal dimensionality while preserving the information significant for the detection task or building methods robust to acquisition noise. Recurrence analysis has been used for identifying transient patterns in experimental EEG (Shalbaf et al., 2015), for classifying patients based on EEG time series (McCarthy et al., 2014) and for prediction of responses during anesthesia (Huang et al., 2006). A key feature of recurrence analysis is to identify sequential states in a multi-dimensional signal space, as shown in most previous studies (beim Graben and Hutt, 2013, 2015). If the experimental data under study is multi-dimensional, for instance a multi-channel EEG recording, the data serves directly as the input to the recurrence analysis. However, it is not valid to compute recurrence plots in the case of univariate time series and hence the data can not be analyzed directly. Therefore, it is necessary to transform the univariate signal to a multivariate (multi-dimensional) signal. Typically this is done by delay-embedding techniques (Webber and Zbilut, 1994; Iwanski and Bradley, 1998) inspired by Takens' theorem (Takens, 1981). The corresponding embedding dimension and delay time in these techniques are chosen rather independent from the dynamic features of the data since typically these are not known *a priori*.

In neuroscience, patterns occurring in certain frequency bands play distinct roles in neural information processing (Kandel et al., 2000; Schnitzler and Gross, 2005). We argue that this additional knowledge can be taken into account and the present work proposes a novel technique based on time-frequency representations of univariate signals. Here, the signal is transformed into its time-frequency representation of spectral power which spans a new phase space in which the signal trajectory evolves. Hence, one may call this transformation *spectral power embedding* since the new phase space encodes instantaneous power in certain frequency bands. The additional advantage of this approach is that it permits to analyse the recurrence structure of data in selected frequency bands. For completeness, we mention that a signal is fully defined by its instantaneous amplitude and phase.

In this work we propose a new method for the detection of metastable states in univariate neural signals. To obtain statistically significant evidence of recurrence structures in signals, we conduct a statistical test over the set of novel, frequency-selective recurrence plots (RP). Below we describe methodologies for building such frequency-selective RPs and performing statistical inference tests. These tests indicate how stable the recurrence plots are with respect to trial-to-trial variability. This novel statistical evaluation is necessary in the analysis of neurophysiological data, since trial-to-trial variability is a well-known experimental finding in such signals. In our work we analyse synthetic transient oscillations and one state variable of the Lorenz attractor involving acquisition noise to validate the methodology. Finally, the study of experimental Local Field Potentials obtained in partially anesthetized ferrets (*Mustela putorius furo*) during a visual stimulus experiment allows to extract new insights into neural information processing. For instance, we show that temporal recurrence occurs in the α-frequency band but not in the δ-frequency band. This result suggests that in the α-band the brain processes the information step-wise (*state by state*) while no step-wise process is performed in the δ-band.

## 2. Materials and methods

In this section we introduce the novel method for studying temporal recurrences common in recurrence plots of different trials. Section 2.1 introduces classical recurrence plots and describes corresponding parameters. Then, we provide a novel method to compute recurrence plots from their time-frequency representations. In Section 2.2 we propose the statistical test method that analyses the similarity of RPs and finds their statistically significant parts. Finally, in Section 2.3 we describe the datasets used in this work.

### 2.1. Recurrence plots and novel time-frequency representations

Recurrence is a fundamental property of dynamical systems which characterizes the behavior of the system in phase space (Poincaré, 1890). A recurrent signal instance is a moment in time when the trajectory returns to a neigborhood of a location in phase space it has already visited previously.

Deterministic dynamical systems are described by their trajectory. A trajectory *x*(*t*) ∈ ℝ^*n*^, *t* ∈ ℝ is sampled at times *t* = *i*Δ*t*, *i* ∈ {1, 2, …, *N*}, where Δ*t* is the sampling time interval and *N* is the total number of samples. For notation simplicity, in this paper we denote the signal sample *x*(*i*Δ*t*) by *x*(*i*). Then, a recurrence plot (RP) is defined as the *N* × *N* matrix **R**, whose elements *r*_*i, j*_ take values *r*_*i, j*_ = 1 when two trajectory samples lay within the open ball B(ϵ) of radius ϵ

(1) $$r_{i,j}=\{\begin{array}{ll} 1, & \text{ if }d(\underline{x}(i),\underline{x}(j))<\epsilon ,\\ 0, & \text{ otherwise,} \end{array}$$

where *d*(·, ·) is a distance function and *i, j* ∈ {1, …, *N*}. Hence, recurrence plots are two-dimensional binary matrices obtained by distance based thresholding and its elements take values *r*_*i, j*_ ∈ {0, 1}. In this work, pixels in RPs will be color-coded white for values *r*_*i, j*_ = 0 and black otherwise.

For an arbitrary chosen ϵ value we can not guarantee that some of the significant dynamic features are not discarded by thresholding. To minimize such a thresholding error, we compute the optimal threshold value ϵ^*^ which maximizes the symbolic entropy for a given distance function, as proposed in beim Graben and Hutt (2013). In more detail, under the assumption that recurrence domains are uniformly distributed for a given recurrence plot, the method constructs disjunct and transitive symbolic recurrence plot matrices from multivariate data. This method permits to identify MSs in a recurrence plot and maps each state (and the transients between the states) to a symbol. Consequently, one maps the high-dimensional dynamics of the system to a sequence of symbols. Let *p*_*k*_ be the probability of the occurrence of the state *k*, i.e., the number of the occurrences of the symbol *k* divided by the total number of occurrences of all symbols. Then maximizing the entropy

(2) $$\begin{array}{l} H\left(\epsilon \right)=-\frac{1}{S_{k,\epsilon }}\overset{S_{k,\epsilon }}{\underset{k=1}{\sum }}p_{k}\log\left(p_{k}\right), \end{array}$$

for a range of ϵ-values yields that value of ϵ for which the distribution of occurrence probabilities {*p*_*k*_} approaches uniformity, i.e., for which all states are equally probable. Here, *S*_*k*, ϵ_ is the number of states for a given ϵ. Then the optimal value

$$\begin{array}{l} \epsilon^{*}=\arg\underset{\epsilon }{\max}H\left(\epsilon \right) \end{array}$$

maximizes the entropy of the extracted symbolic sequence and hence the recurrence structure of the data. This optimal value is computed for each dataset separately.

After defining conventional RPs and computation of the optimal parameter ϵ, the remaining part of this section focusses on how to build frequency-selective recurrence plots. Many biophysiological signals have characteristic frequency signatures. For example, the human heart beats about sixty times per minute in average, i.e., at the frequency of 1 Hz. Another example are eye blinks that induce signal changes in the α-frequency band (frequencies in the interval 8–12 Hz) in EEG recordings. To take into account the distinct signatures of spectral bands present in neural signals, we propose a novel concept for building recurrence plots from time-frequency signal representations, instead of building them directly from univariate data or constructing them by employing delay-embedding techniques. Such representations, in general adapted for non-stationary signal analysis, give insights into frequency bands of importance and provide additional flexibility to recurrence plot analysis that is not present in time-domain, for e.g., the possibility to weight the importance of some frequency bands. In the literature there are several ways to choose values of the frequency bands. We use the following frequency interval definitions: the δ-frequency band denotes the interval [0.5 Hz; 4 Hz], the θ-frequency band the interval [4 Hz; 8 Hz], α-band [8 Hz; 12 Hz], β-band [12 Hz; 20 Hz], and the γ-band denotes the interval [20 Hz; 40 Hz].

**Figure 1:**
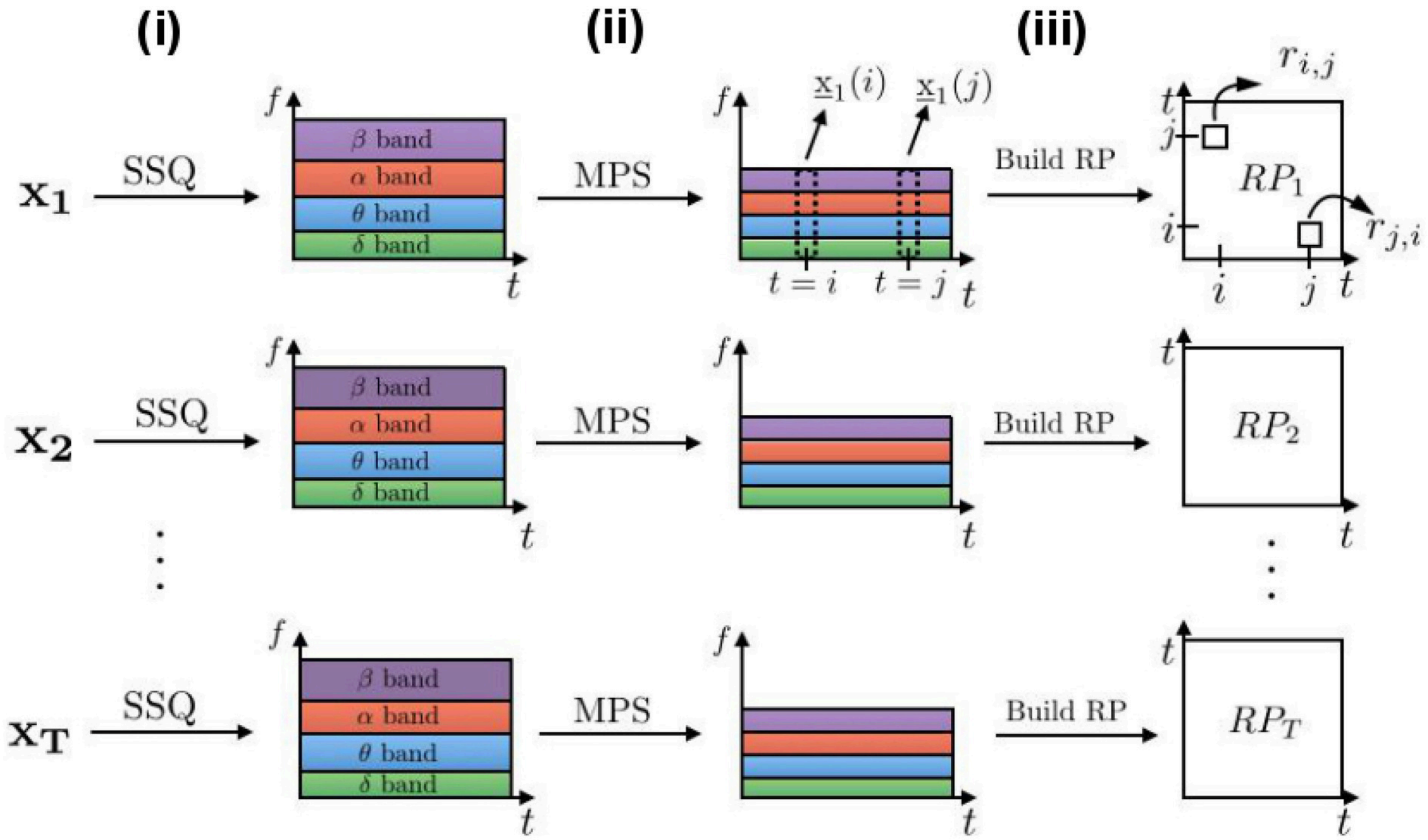
**Building frequency-selective recurrence plots from ***T*** time series**. Processing blocks are represented by arrows: **(i)** SSQ is a synchrosqueezing transform block used to obtain time-frequency representations of signals; **(ii)** the MPS block computes mean values of the power spectrum for each of the chosen frequency bands, which reduces the signal dimensionality; **(iii)** This signal is the basis for the recurrence analysis leading to recurrence plots ${\left\{RP_{k}\right\}}_{k=1}^{T}$.

We build novel recurrence plots in three steps, as shown in Figure 1: (i) we expand the set of *T* univariate trials {**x**_1_, **x**_2_, …, **x**_*T*_} to their corresponding time-frequency domains; (ii) we compute the mean power of the spectrum over certain sets of frequencies. These mean power time series may be called $y_{j}(t)\in {\mathbb{R}}^{S},\;j=1,\ldots ,T$, where *S* is the number of frequency bands; (iii) we compute recurrence plots by computing distances between vectors *y*(*i*) and *y*(*j*), *i, j* ∈ {1, …, *N*} as in Equation (1). In the following paragraphs we describe these blocks in more detail.

The first block in Figure 1 provides a time-frequency representation of the signal. In classical spectrogram calculations, the stronger (weaker) is the localization of signals in time, the larger (smaller) are their localization windows in frequency. This effect is called the uncertainty principle implied in the Fourier transform. A synchrosqueezing (SSQ) transform overcomes this deficiency by performing wavelet-based filtering and signal power reassignment to the appropriate frequencies. In addition, Meignen et al. (2012) and Auger et al. (2013) show that SSQ is superior for processing neural signals when compared to conventional spectral analysis methods, such as continuous wavelet transform or spectrogram. Hence, we use the SSQ transform defined in Section 2.1.1 as the processing block (i) in Figure 1.

The second block in Figure 1 computes the mean value of the power spectrum (MPS) for sets of frequencies, see Section 2.1.2 for details. This is one of the basic features for studying neural signals. We assume that the dynamics of the neural system encoded in frequencies is proportional to the power spectrum in sets of frequencies. This analysis step provides multi-variate time series whose dimension is equal to the resulting vector of averaged frequency bands.

Finally, in the third processing block in the figure we compute recurrence plots from the obtained time-frequency dataset as in Equation (1). If we do not explicitly mention otherwise, we use features from all the frequency bands to compute recurrence plots. In the experimental ferret dataset, we additionally present cases when recurrence plots are calculated from the single frequency band features such as δ- or α-frequency bands, since these bands play an important role in the loss of consciousness under anesthesia.

To summarize, the proposed method for building recurrence plots from time-frequency representations grasps band-related features and allows flexibility in the analysis of particular frequency bands, which is not possible in the classical RP analysis. Our approach however requires additional computations of the synchrosqueezing transform and mean power of the spectrum.

#### 2.1.1. Synchrosqueezing transform

For completeness of this work, in this section we provide the mathematical definition of the synchrosqueezing transform (Meignen et al., 2012; Auger et al., 2013), that we use as a processing block in the proposed algorithm, see Figure 1. We presume that input signals are composed of several components with time-varying oscillatory characteristics. In other words, we assume that signals *f*(*t*) can be well approximated with *K* signal components, $f\left(t\right)=\overset{K}{\underset{k=1}{\sum }}f_{k}\left(t\right)+e\left(t\right)$, $f_{k}\left(t\right)=A_{k}\left(t\right)e^{2\pi i\phi_{k}\left(t\right)}$, where *A*_*k*_(*t*) and $\phi_{k}^{{}^{\prime }}\left(t\right)=\frac{1}{2\pi }\frac{d\phi_{k}\left(t\right)}{dt}$ denote the amplitude and the instantaneous frequency (IF) of each component and *e*(*t*) represents a small error. We assume that the components *f*_*k*_ have slowly time-varying amplitudes *A*_*k*_(*t*) and sufficiently smooth IFs. These conditions assure that signal components are well separated in frequencies and the complete definition is available in Thakur et al. (2013), Def. II.1 (codes available online in Thakur, 2013).

Let a wavelet ψ(*t*) be a square integrable and normalized function. Then, its scaled and time-shifted variants $\psi \left(\frac{t-b}{a}\right)$ represent a set of scaled bandpass filters. In the following, we denote the frequency of one signal component by $\omega_{k}\approx 2\pi \frac{d\phi_{k}\left(t\right)}{dt}$. A Continuous wavelet transform (CWT) of the function *f* at scale *a* and time shift *b* is defined by $W_{f}\left(a,b\right)=\frac{1}{\sqrt{a}}\int_{-\infty }^{\infty }f\left(t\right)\bar{\psi \left(\frac{t-b}{a}\right)}dt$, which represents a convolution of scaled and band-passed filters with the signal. The shifts of wavelet function are driven by the scale value *a*. For example, for the first signal component with frequency ω_1_, the value of the wavelet coefficient *W*_*f*_(*a*_1_, *b*) spreads around the scale factor $a_{1}=\frac{\omega_{\psi }}{\omega_{1}}$, where ω_ψ_ is the central wavelet frequency. Therefore, the estimated IF in the neighborhood of this value of the scale is equal to the frequency ω_1_. The synchrosqueezing transform *T*(ω_*q*_, *b*) uses estimates of the instantaneous frequency ω_*f*_(*a, b*), computed for each scale-time pair (*a, b*) by $\omega_{f}\left(a,b\right)=-iW_{f}\left(a,b\right)\frac{\partial W_{f}\left(a,b\right)}{\partial b}$ to reallocate the energy of the wavelet coefficients. Let Δ*a*_*p*_ (Δω) denote resolution steps in scale (frequency). Then, this transform, defined by $T\left(\omega_{q},b\right)=\underset{a_{p}:|\omega_{f}\left(a_{p},b\right)-\omega_{q}|\leq \Delta \omega ∕2}{\sum }W_{f}\left(a_{p},b\right)a^{-3∕2}\Delta a_{p}$ enhances frequency localization of oscillating components of the signal and provides more precise time-frequency representations of the signal. In analogy to the spectrogram used in classical short-time Fourier analysis, we plot values

(3) $$\begin{array}{r} S\left(\omega_{q},b\right)=|T\left(\omega_{q},b\right){|}^{2} \end{array}$$

for each pair (ω_*q*_, *b*) in time-frequency plots, see **Figures 5A,E**, **6A,C**.

#### 2.1.2. Mean power spectrum

For each frequency band with *Q* components, the mean power spectrum value is defined by

(4) $$\begin{array}{r} MPS\left(t\right)=\frac{1}{Q}\overset{Q}{\underset{q=1}{\sum }}S\left(\omega_{q},t\right), \end{array}$$

where *S*(ω_*q*_, *t*) is defined in Equation (3), ω_*q*_ are frequencies of one frequency band and *t* is time.

### 2.2. Statistical analysis

We study statistical properties of frequency-selective RPs obtained from time-frequency trial representations. By virtue of noise effects and an expected trial-to-trial variability, recurrence plot structures are expected to vary from trial to trial. To evaluate the recurrence plots statistically, we perform a statistical inference analysis based on a classical chi-squared test (Yates, 1934). To this end, we construct surrogate recurrence plots and employ an inference test.

Classically, surrogate sets of univariate signals (Schreiber and Schmitz, 2000) preserve some of the important features of the original time series, for example the spectrum magnitude, while they replace the phase values by a random sequence of values. The reasoning behind this randomization is that time domain reshaping destroys non-stationarities, so the local spectral components will vary while the global spectrum remains the same. As a consequence, the mean and variance of the signal do not change (Borgnat et al., 2010; Richard et al., 2010).

**Figure 2:**
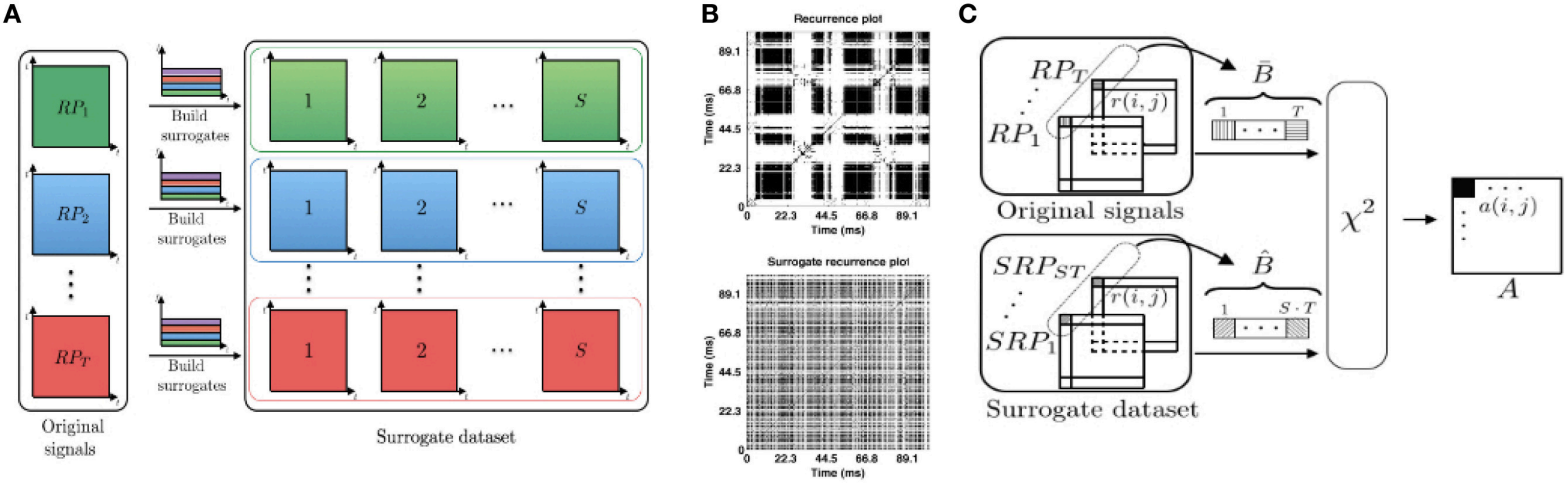
**(A)** Surrogate set construction: for each of *T* time-frequency signal representations used for computing the original set of RPs, one computes *S* surrogate RPs. The resulting *S* surrogate recurrence plots have the same energy in time-frequency domain. In total, there are *S*·*T* surrogates. **(B)** Examples of the RP (top) and the surrogate RP (bottom) for the transient oscillations dataset. **(C)** Illustration of the pixel-wise χ^2^ statistical test, more details in the text.

In this work, we build the surrogate dataset with the same power spectrum as in the original data, where the information component encoded in time is randomized, cf. Figure 2A. For each time index of the signal we randomly select a novel index value, such that all the index values are chosen exactly once (permutations without repetition). Then, we rearrange the time-frequency representation of trials according to the chosen index values and compute recurrence plots of surrogates by repeating steps (ii) and (iii) shown in Figure 1. This procedure is repeated *S* times per trial. Figure 2A illustrates how to obtain the surrogate set from *T* trials. Examples of an original RP and a corresponding surrogate RP are provided in Figure 2B.

We compare pixel-related statistical measures between the set of the original recurrence plots from different trials and their surrogates to determine whether original RPs preserve the common underlying signal dynamics in statistically significant way. This comparison is illustrated in Figure 2C. In detail, we denote the set of *T* recurrence plots obtained from the original trial data by ${\left\{RP_{k}\right\}}_{k=1}^{T}$ and its surrogate set by ${\left\{SRP_{k}\right\}}_{k=1}^{S\cdot T}$. In our simulations, there are *T* = 10 trials in total, where the number of surrogates generated per trial is *S* = 100. The full set of surrogates counts *S*·*T* = 1000 surrogate RPs. At first, we perform *pixel-wise* statistical analysis tests between the corresponding pixels of the original and the surrogate recurrence plots. Let $\bar{B}={\left\{r_{i,j}^{\left\{k\right\}}\right\}}_{k=1}^{T}$ be the vector that consists of the set of pixels with same coordinates in the original RPs and $\hat{B}={\left\{r_{i,j}^{\left\{k\right\}}\right\}}_{k=1}^{S\cdot T}$ is the corresponding vector of pixel values for surrogates. Vectors $\bar{B}$ and $\hat{B}$ consist of values from the set {0, 1}, since RP elements *r*_*i, j*_ by definition take binary values, cf. Equation (1). To perform a chi-square test for categorical data, we build a two-by-two contingency table. For explanation, this tables first row takes values from the original RPs and the second row contains values from surrogate RP. The first table column marks the number of values *r*_*i, j*_ = 1 and the second column the number of elements *r*_*i, j*_ = 0. The elements of this table (two rows and two columns) have the coordinates (*l, m*), *l, m* ∈ {1, 2}. Then, the chi-square statistics for the pixel (*i, j*), *i, j* ∈ {1, …, *N*} is computed by

$$\begin{array}{l} \chi^{2}\left(i,j\right)=\underset{l\in \left\{1,2\right\}}{\sum }\underset{m\in \left\{1,2\right\}}{\sum }\frac{{\left(f_{o}^{\left(i,j\right)}\left(l,m\right)-f_{e}^{\left(i,j\right)}\left(l,m\right)\right)}^{2}}{f_{e}^{\left(i,j\right)}\left(l,m\right)}\;. \end{array}$$

Here, $f_{o}^{\left(i,j\right)}\left(l,m\right)$ is the observed table value at the coordinate (*l, m*) for the pixel (*i, j*) and $f_{e}^{\left(i,j\right)}\left(l,m\right)$ is its expected frequency. The latter value is computed as $f_{e}^{\left(i,j\right)}\left(l,m\right)=n_{r}\left(l\right)n_{c}\left(m\right)∕q$, where *n*_*r*_(*l*) is the total number of elements in the row *l*, *n*_*c*_(*m*) is the number of elements in the column *m* and *q* is the total number of elements in the two-by-two table. The calculated chi-square value is compared with the result in the chi-square table for predefined values of the degree of freedom *df* = 1 and the significance level α_*s*_ = 0.05. If the calculated chi-square value is larger than the value in the table, the hypothesis that signals share the same distribution is rejected, see Yates (1934) for more details. In this work, the outcomes of chi-square tests are visually represented as matrices *A* = [*a*_*i, j*_], *i, j* ∈ {1, …, *N*} whose elements take values

(5) $$a_{i,j}=\{\begin{array}{ll} 1,\text{​} & \text{if distributions of trial and surrogate sets}\\ & \text{are different},\\ 0,\text{​} & \text{otherwise}. \end{array}$$

In this work all the figures follow the same color code as for illustrating recurrence plots, i.e., white pixels denote values *a*_*i, j*_ = 0 and black pixels stand for *a*_*i, j*_ = 1.

Since single elements in RPs are correlated to neighboring elements caused by the underlying dynamics, the underlying assumption of independent recurrence matrix elements does not hold and corrections of the significance test should be applied, such as the Bonferroni correction. To this end, in the examples of artificial datasets, we have performed a *t*-test which is based on the hypothesis that original and surrogate signals have the same distribution of mean values. The statistics are computed based on the pixels and their mean values in a 5 × 5 neighborhood around each pixel. In addition, we have applied a Bonferonni correction.

### 2.3. Datasets

**Figure 3:**
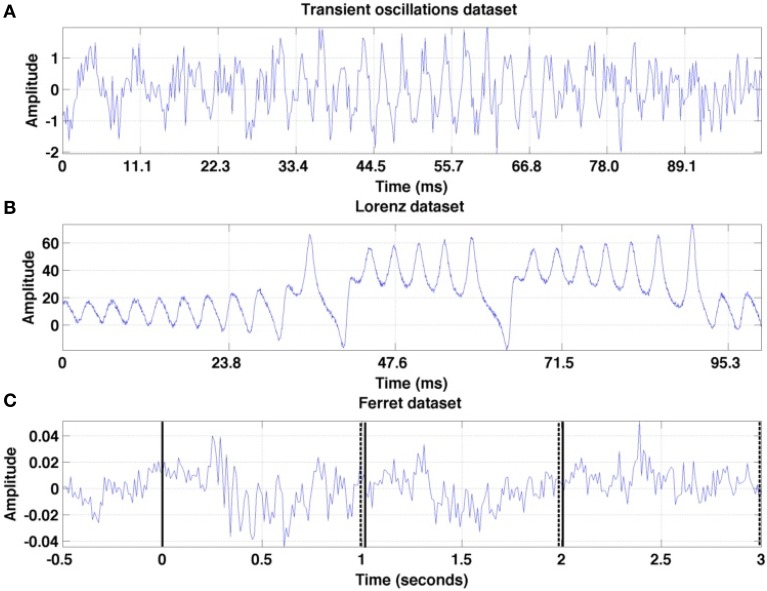
**Illustration of the first trial in each of the three time series under study**. **(A)** Transient oscillations **(B)** Lorenz attractor **(C)** Ferret dataset: trial from the set *session one* is recorded at a granular layer electrode. The vertical solid lines denote the stimulus onsets and the set of dashed lines mark the stimulus offsets.

To illustrate different analysis steps and to validate the power of the proposed method, we first apply the proposed algorithm to two artificial datasets. Then, the methodology is applied to experimental datasets. Single trials of these datasets are illustrated in Figure 3 and their origin is described in detail below. For both artificial datasets, we model the tria-to-trial variability by a temporal shift of the data in time combined with additive measurement noise.

#### 2.3.1. Transient oscillations

A modified Lotka-Volterra model with *n* = 3 interactive elements (Rabinovich et al., 2008a,b)

(6) $$\begin{array}{r} \frac{dx_{i}\left(t\right)}{dt}=x_{i}\left(t\right)\left(\sigma_{i}-\overset{n}{\underset{j=1}{\sum }}\rho_{i,j}x_{j}\left(t\right)\right), \end{array}$$

serves as an abstract model of event-related brain potentials (beim Graben and Hutt, 2015). Here *x*_*i*_(*t*) ≥ 0, *i* ∈ {1, 2, 3} is the activity rate of the element *i*, σ_*n*_ is the growth rate of the n-th population and ρ_*i*_ define interactions between elements. In our setup, σ_1_ = 1, σ_2_ = 1.2 and σ_3_ = 1.6, ρ_*ii*_ = 1, ρ_12_ = 1.33, ρ_13_ = 1.125, ρ_21_ = 0.7, ρ_23_ = 1.25, ρ_31_ = 2.1, and ρ_32_ = 0.83. The output signal *s*(*t*) is a linear superposition of transient oscillations with frequencies ν_1_ = 170 Hz, ν_2_ = 20 Hz, ν_3_ = 75 Hz, where at one time instance, only one of these three components is dominant, see more details below. We point out that these frequencies are chosen rather arbitrarily for an optimal illustration. The activity rate *x*_*i*_ defines the amplitude of the component *a*_*i*_ with frequency ν_*i*_ and the output signal obeys

(7) $$\begin{array}{r} s\left(t\right)=\overset{3}{\underset{i=1}{\sum }}a_{i}\left(t\right)\sin\left(2\pi \nu_{i}t\right)+\xi \left(t\right)\;,\;a_{i}\left(t\right)=e^{-{\left(x_{i}-\sigma_{i}\right)}^{2}∕2\eta_{i}^{2}}, \end{array}$$

with η_1_ = 0.5, η_2_ = 0.33, η_3_ = 0.4. By this construction, the amplitudes *a*_*i*_ increase and decrease in a certain time window outside of which they almost vanish. These windows of the three oscillation modes *i* = 1, 2, 3 do not overlap and the transitions between them are rather rapid. The variable ξ(*t*) represents measurement noise and its random values are i.i.d. Gaussian noise with zero mean and variance 0.5. The sampling rate is 450 Hz. We generate 10 trials which are time-jittered by shifting the trials by 1 sample to later instances, while each trial is subject to additive noise different in each trial. A single trial is given in Figure 3A.

#### 2.3.2. Lorenz dataset

The Lorenz system (Lorenz, 1963) is a well-studied three-dimensional differential equation system

$$\begin{array}{l} \frac{dx}{dt}=-\sigma x+\sigma y\;,\;\frac{dy}{dt}=\rho x-y-xz\;,\;\frac{dz}{dt}=-\beta z+xy \end{array}$$

with σ = 10, ρ = 28, β = 8∕3. Its solutions show non-trivial transient dynamics and their wings represent metastable states as explained above (cf. beim Graben and Hutt, 2013). We study the univariate time series *x*(*t*), which is the solution of the above given system of equations. This time series may represent a macroscopic measured signal such as EEG recording (Skarda and Freeman, 1987; Basar, 2006), capturing activity from different metastable sources. The sampling rate is equal to 2100 Hz. We generate ten signal trials time-jittered by shifting the signal by 1 sample to later instances and add i.i.d. zero mean Gaussian noise with unity variance to the signals. One trial signal is illustrated in Figure 3B.

#### 2.3.3. Ferret dataset

**Figure 4:**
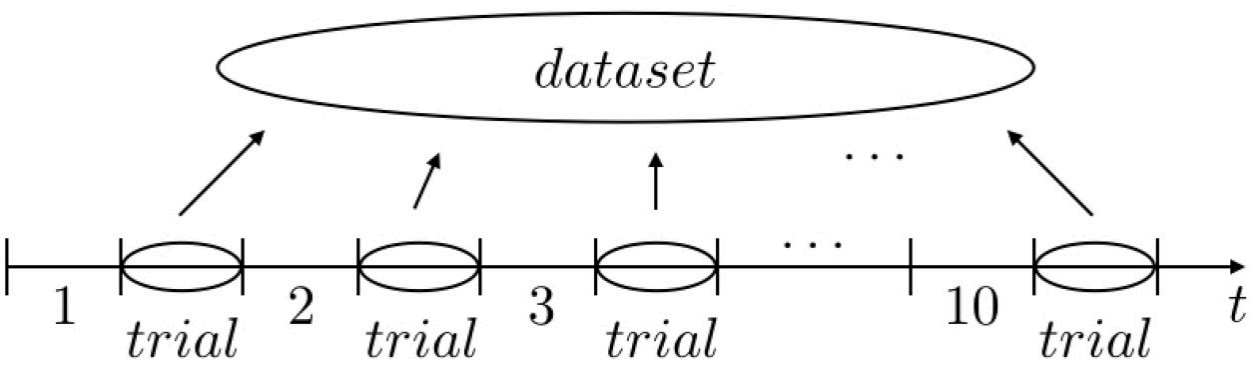
**Experimental paradigm of the ferret experiment**. In the original recording protocol (Sellers et al., 2013, 2015a,b), 10 visual stimuli are interleaved by several types of different stimuli, represented by numbers in the figure. Our dataset consists of the responses to sine-wave luminance gratings only. Other types of stimuli are the black screen (intervals marked by {4, 6, 9}), checkerboard noise for {2, 7} or fox images stimuli. The latter set consists of the weakly spatially filtered image of foxes in the intervals marked by {3, 5} and strongly spatially filtered image of foxes in the interval set {1, 5, 10}.

The experimental dataset under study in the present work are Local Field Potential (LFP) measurements collected as described in Sellers et al. (2013, 2015a,b). Briefly, female ferrets were anesthetized, intubated, and underwent surgery to gain access to primary visual cortex (V1, ~3 mm anterior to lambda and 9 mm lateral to the midline). Anesthesia induction was achieved with an intramuscular injection of ketamine (30 mg/kg) and xylazine (1–2 mg/kg), and anesthesia maintenance was achieved with 1.0% isoflurane (10–11 cc, 50 bpm, 100% medical grade oxygen), with continuous IV infusion of xylazine (1.5 mg/kg/h xylazine with 4.25 mL/h 5% dextrose lactated ringer's). Animals were head-fixed in front of the presentation screen and a 32-channel depth probe was acutely inserted into cortex (50 microns contact spacing along the z-axis, NeuroNexus, Ann Arbor, MI) and was positioned to cover all cortical layers. The reference electrode was located on the same shank (0.5 mm above the top recording site) and was positioned in 4% agar in saline above the brain. The full-field visual stimulus was presented on a 52 × 29 cm monitor with 120 Hz refresh rate and full high-definition resolution (1920 × 1080 pixels, *GD*235 Hz, Acer Inc, New Taipei City, Taiwan) at 47 cm distance from the animal. Each trial was 30 s long and consisted of three parts: (i) recording interval [0−10)s is a baseline (screen is black); (ii) *t*_*s*_ ∈ [10−20) s is the presentation of the sine-wave luminance gratings; (iii) [20−30) s is “post-baseline” (screen is again black). Visual stimuli were interleaved with other types of stimuli (all in randomized order), for instance with a black screen or with a strongly spatially filtered image of foxes (foxes are natural enemies of ferrets), see Figure 4. The sine-wave luminance grating was presented at a rate of 1 Hz for 10 s (during each 1 s period, progressive frames transitioned from black to white to black and all the screen pixels had the same color for any given frame). In the subsequent analysis, we consider a subset of recordings. The dataset under study starts 0.5 s before stimulus onset and lasts until 3 s at the end of the third stimulus cycle. This stimulus is a black screen at *t* = 0, 2, and 3 s with luminance maxima at *t* = 0.5, 1.5 s, and 2.5 s. A single trial is illustrated in Figure 3C.

Electrophysiological recordings were conducted during stimulus presentation. Unfiltered signals were first amplified with MPA8I head-stages with gain 10 (Multichannel Systems, Reutlingen, Germany), then further amplified with gain 500 (Model 3500, A-M Systems, Carlsborg, WA), digitized at 20 kHz (Power 1401, Cambridge Electronic Design, Cambridge, UK), downsampled to 1 kHz afterwards, and digitally stored using Spike2 software (Cambridge Electronic Design). In total, 20 trials across two sessions conducted on different days were analyzed, the session sets of 10 trials are called *session one* and *session two* in the following. Datasets are downsampled to the sampling rate equal to 100 Hz. All procedures were approved by the University of North Carolina-Chapel Hill Institutional Animal Care and Use Committee (UNC-CH IACUC) and exceed guidelines set forth by the National Institutes of Health and U.S. Department of Agriculture.

## 3. Results

In this section, we first apply the proposed method to two artificial datasets to verify if it reveals dynamics given the noisy set of trials. After validation of the method, we apply it on the experimental dataset (ferret dataset) and study whether it well extracts the dynamics from the recorded trials.

To understand the results, we first shortly describe recurrence plots for several simple test signals. As previously mentioned, black pixels denote recurrence events and white ones its absence. All recurrence plots have a black diagonal line, by definition (see Equation 1). Signals without any recurrence have a white square RP with a black diagonal line. Random noise signals have a random distribution of black pixels in the plot, with the exception of the black diagonal line. A simple periodic signal has a recurrence plot that consists of the black diagonal line and other black lines that are parallel to the diagonal, where the distance between them will reveal the period of the signal. More complex signals that have recurrent states may show different structures in RPs, for example, checkerboard-like patterns. These black colored fields, to which we refer as to recurrence domains, may have different sizes and shapes. For two artificial datasets we expect to observe repetitive black patterns that correspond to repetitive states within signal components. For the experimental dataset, we expect to observe recurrence patterns that are directed by the onset of the visual stimulus.

### 3.1. Artificial datasets

**Figure 5:**
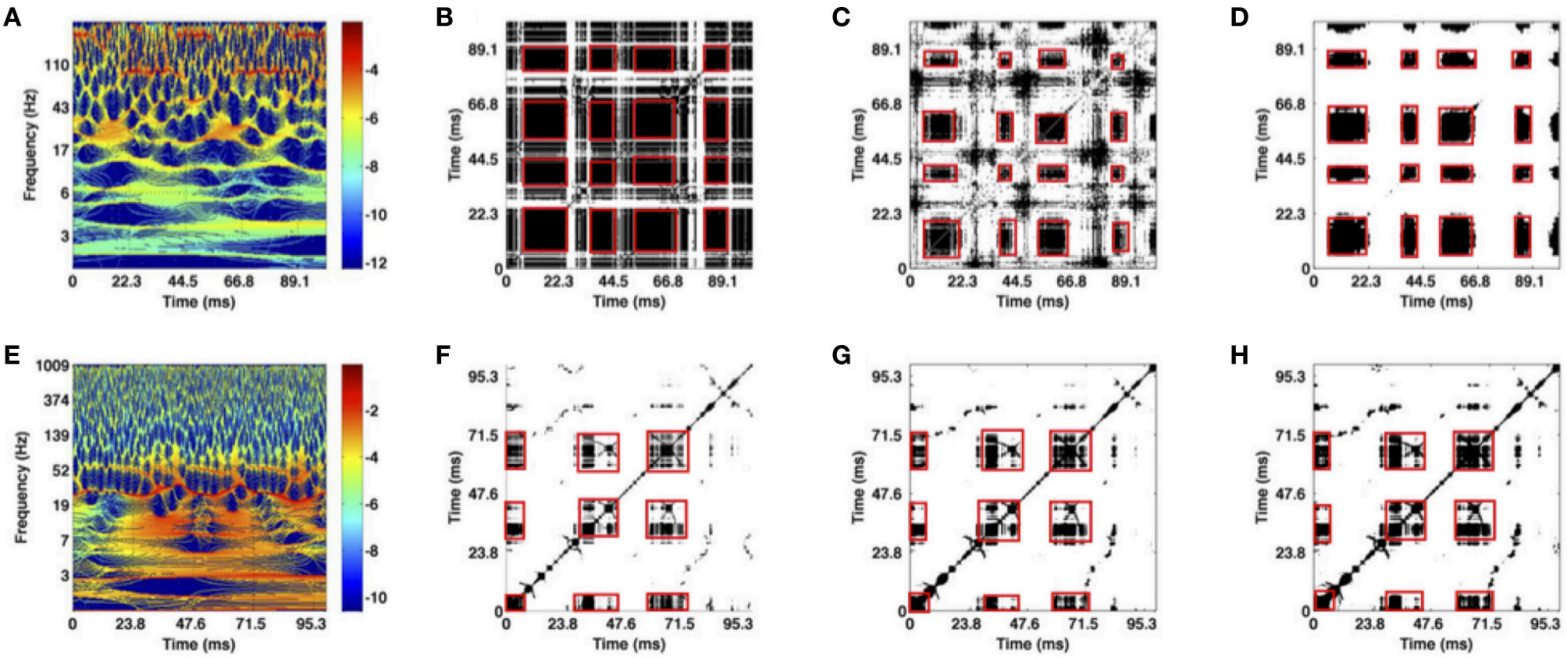
**Illustration of time-frequency (A,E), optimal recurrence plot (B,F) for the trials of two artificial datasets shown in Figures 3A,B, statistically significant recurrence plots (C,G) based on 10 trials and classical ***t***-test results (D,H)**. Values plotted in black represent areas where the original and the surrogate set differ significantly (rejected test). **(A–D)** Transient oscillations; **(E–H)** Lorenz attractor. The red boxes mark recurrent metastable states.

We demonstrate our methodology in Figure 5, which shows the analysis steps for the examples of transient oscillations (Figures 5A–D) and the Lorenz attractor (Figures 5E–H).

The time-frequency representation of one transient oscillation trial is shown in Figure 5A. As previously mentioned in Section 2.3.1, the corresponding signal exhibits three periodic components. We visually inspect the figure and observe high power spectrum values around the following time windows: (i) {(0, 11), (42, 54), (85, 97)} ms around ν_1_ = 170 Hz (dark red horizontal line segments); (ii) {(19, 26), (64, 72)} ms around ν_2_ = 20 Hz (broad orange areas); (iii) {(24, 38), (74, 87)} ms around ν_3_ = 75 Hz (dark red horizontal line segments). Note that for other trials these values may fluctuate because the frequency and time window of the current active component vary due to noise that models trial-to-trial variability. Figure 5B shows recurrent blocks (in black) in a single trial which correspond well to the dynamics observed in the data, cf. Figures 5A, 3A. For explanation, these recurrence blocks correspond to MSs and the white parts represent transients between them.

The time-frequency representation of one Lorenz attractor trial is given in Figure 5E. The approximate time intervals during which the system stays in each of the two wings are visually inspected from the power spectrum values. For the wing in time intervals {(0, 30), (90, 100)} ms, Figure 5E shows a peak at ~30 Hz corresponding to the oscillation frequency in the Lorenz wing, see Figure 3B. The other wing is reached in the time intervals {(40, 60), (65, 80)} ms in accordance to the power peak at about 40 Hz. Note that for other trials time intervals may be different due to varying trials in the set. Figure 5F shows recurrent blocks in a single trajectory. The recurrence blocks repeat in the correct time windows and represent the different wings, i.e., the MSs.

Time-frequency representations of single trials are the basis for the recurrence analysis leading to recurrence plots given in Figures 5B,F for the respective datasets. These plots show the metastable dynamics of the transient oscillations and the Lorenz trajectories in the corresponding time windows as recurrent structures. The recurrent, i.e., repetitive, structure is visible in the illustrated trial of the corresponding data. Now, considering several trials these recurrent structures may vary due to the trial-to-trial variability. Nevertheless, to study the recurrent structure *common* to all trials, we employ the statistical inference method and extract statistically significant areas of recurrence plots, as shown in Figures 5C,D,G,H. The recurrent structure is obvious in these plots, reflecting the underlying recurrence structure in the artificial signals. In addition, these results demonstrate that the methodology extracts recurrence structures common in several trials, although the recurrent structure is less obvious in single trials, Figures 5B,C. Figures 5D,H show the multiple comparison-test results for both artificial datasets. The white area increases and the black areas are more focussed on the red squares, i.e., spurious recurrences (black dots) are removed and and separated well from transient (white areas). Hence the multiple-comparison test improves the statistical inference.

We point out again, that the extraction of the recurrent structure from the univariate data shown in Figure 5 is possible only by the spectral power embedding, i.e., the transformation of the univariate data into multivariate data. The subsequent preliminary statistical inference allows to identify the recurrent MSs which are common in all trials with a confidence of 0.95. By virtue of the spectral power embedding, the method permits to select certain frequency bands to study recurrence structure in specific frequency bands. This new element renders the spectral power embedding more flexible and hence superior to previous embedding techniques, such as the delay embedding based on Takens theorem. To illustrate this, the subsequent section shows results from experimental data in different frequency bands.

### 3.2. Experimental data

After studying artificially generated trials and verifying that the proposed method extracts well the dynamics features given by repetitive black structures, we now investigate whether such structures can be found in the experimental data as well.

**Figure 6:**
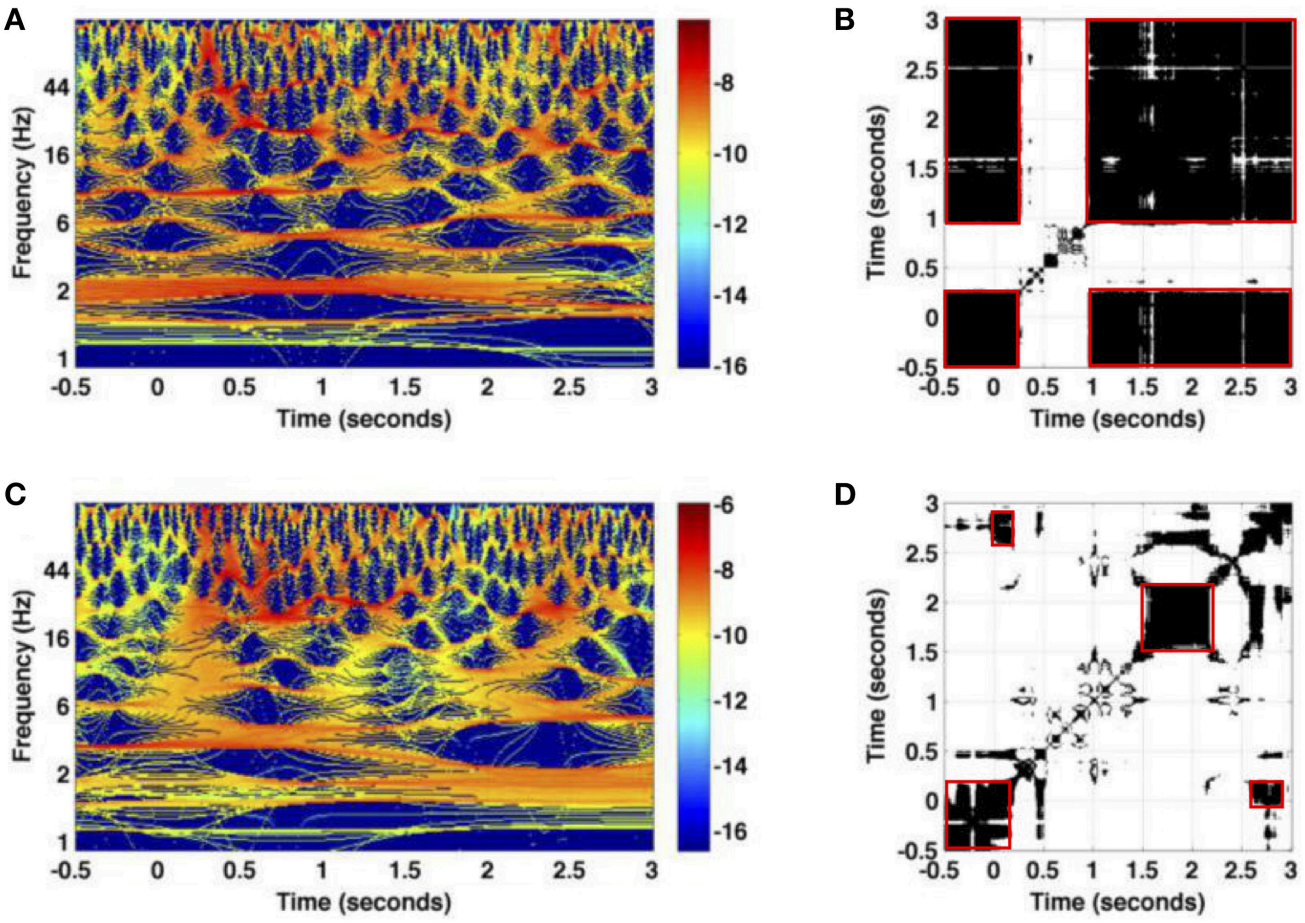
**Illustration of trial variability for the experimental dataset ***session one*****. **(A,C)** The logarithm values of the power spectrum for two trials measured at an electrode in the granular layer; **(B,D)** the corresponding recurrence plots. Non-zero values of recurrence plots are encoded in black. Red boxes denote recurrent metastable states.

Figure 6 provides the time-frequency representations of two single trials of the same session Figures 6A,C and the corresponding recurrence plots Figures 6B,D. We observe a high trial-to-trial variability between both trials. This can be observed both in the time-frequency representations and the resulting recurrence plots. For instance, Figure 6B shows a single recurrent state in the data except in the time window during the first stimulus at *t* ∈ [0.25 s;1 s]. Hence the system remains close to the resting state (*t* < 0) during the first stimulus. Conversely, Figure 6D reveals that the baseline activity, i.e., activity before stimulation in the time interval [−0.5 s;0 s], recurres in the interval [2.5 s;3 s]. In addition, the activity at about *t* = 2 s resembles the activity just after *t* = 2.5 s. These different findings for two trials are surprising since the experimental presentation of the visual stimulus is well-controlled and the stimulus is simple enough to expect almost identical neural responses.

**Figure 7:**
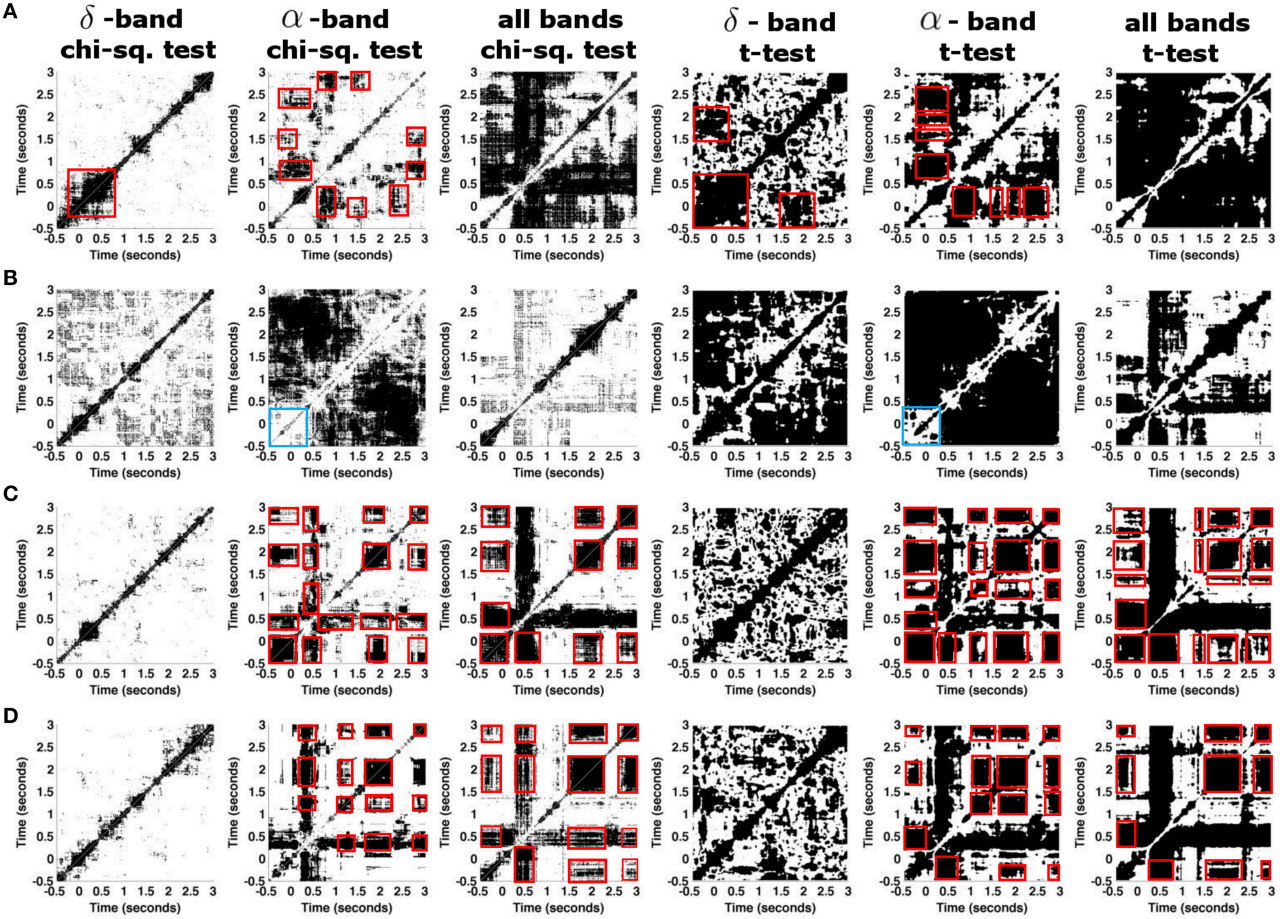
**Statistically significant parts of recurrence plots for different ferret datasets**. **(A)** Results from a dataset of *session one* that consists of 10 trial recordings of the single granular sensor *s*_10_. **(B)** Same as in **(A)** for the dataset *session two*. **(C)** Results are calculated based on 10 averaged trials in dataset *session one*, where each trial is averaged over 8 granular layer time series. **(D)** Same as in **(C)** for dataset *session two*. Red boxes indicate MSs, blue boxes in **(B)** indicate a prominent lack of recurrence in the α−band.

To reveal the recurrent structure that is common in all trials, we now study the trial-to-trial variability of recurrence plots and aim to reveal whether the signal trials preserve the same dynamical behavior, cf. Figure 7. Applying the statistical method, we investigate the similarities of the results obtained from ten trials measured by a single granular sensor and from the set of 10 averaged signal trials, where the average is taken over eight granular layer sensors. This analysis is done for both ferret datasets. Moreover, we detail the analysis considering particular frequency bands which are of interest for anesthesia. To this end, we compute recurrence plots using the values of the power spectrum coefficients in the corresponding frequency bands as illustrated in Figure 1.

Figure 7 shows the statistically significant parts of the recurrence plots for the δ- and the α-frequency band and for all frequencies (chi-square and *t*-test results). The figure reveals that there is no statistically significant recurrent structure in the δ frequency band in signals under study. Conversely, the α-frequency band exhibits significant recurrent structures in the single granular electrode in both datasets, cf. Figures 7A,B. For instance, in *session one* the first response to the stimulus at *t* = 0 s returns at *t* = 1 s. Results for all frequency bands differ to results obtained in the α frequency band. The differences are dependent on the experimental sessions suggesting the presence of strong recurrences in bands different to α and δ or strong noise artifacts. To gain further insights into the dependence on frequency bands, we consider single trials which represent spatial averages of time series from adjacent granular layers. This average denoises the time series. Figures 7C,D shows the corresponding results. Figures 7C,D show results from data in both experimental sessions revealing a similar recurrence structure now. Considering all frequency bands yields recurrences similar to the one obtained in the α-band. Specifically, the prominent cross-shaped structure located at *t* = 0.5 s indicates a MS common to all data with *t* ≥ 0.5 s. Additional recurrences occur in the time intervals [−0.5;0], [1.7;2.2], and [2.7;3.0] ms. These results are consistent in the chi-square significance tests and the *t*-test involving corrections for multiple comparison. At last, we mention the prominent lack of recurrence in the baseline time interval observed in a single electrode in *session one*, cf. Figure 7B. Since it does neither occur in *session one* nor in the spatially averaged data shown in Figures 7C,D, it appears to be spurious and is neglected.

**Figure 8:**
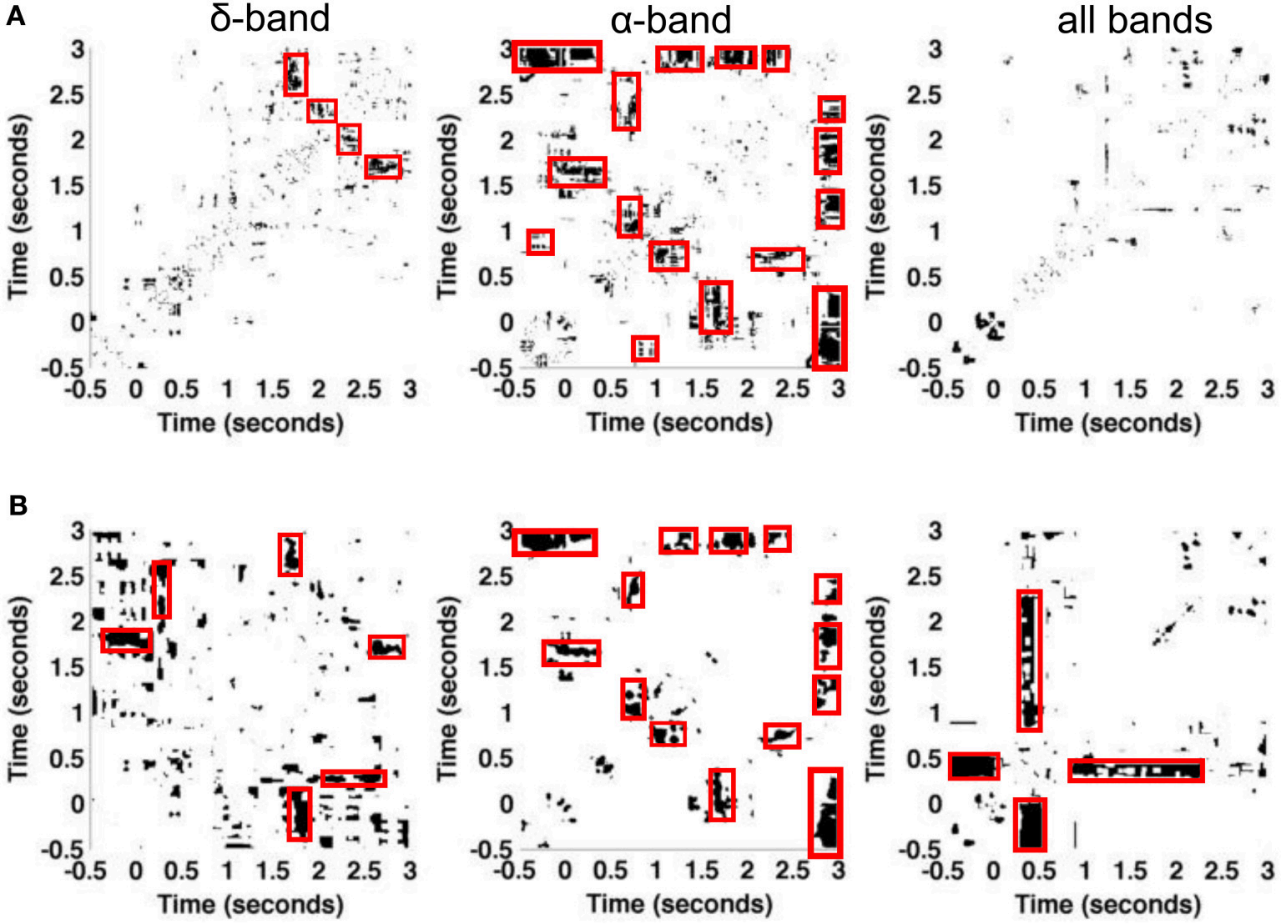
**Illustration of the influence of different visual pre-stimuli on resulting plots in experimental dataset**. Statistically significant areas of recurrence plots are obtained by **(A)** pixel-wise chi-square tests and **(B)** *t*-test between trials with pre-stimulus *black screen* and those whose pre-stimulus is *fox image*. The data is taken from a single granular layer electrode in datasets *session one* and *session two* together. Both the pre-stimulus *black screen* and *fox image* have occurred three times among the 10 trials in each dataset. Significantly different values are coded as white pixels, statistically similar values are coded as black pixels.

The experimental paradigm includes visual presentations of stimulus types in a randomized order. The previous paragraphs show neurophysiological responses to the sine-wave stimulus only. To gain further insight into the trial-to-trial variability subject to various pre-stimuli, we have selected two subsets of sine-wave trials that have two different preceding stimuli, namely the “black screen” subset denoted by *subset one* and the subset of “strongly spatially filtered version of foxes” denoted by *subset two*. *Subset one* includes the trials {4, 6, 9}, while *subset two* is composed of the trials {1, 5, 10} of datasets *session one* and *session two*. The comparison of various pre-stimuli data is done by the chi-squared difference measure based on recurrence plots of both subsets. Figure 8 shows the statistically significant recurrences that are common in stimulus responses on both types of pre-stimuli. The diagonal lines are absent from figures, which suggests that at each time instance two comparison signals differ. Poor but visible recurrent structures in δ-band are grouped into two distinct blocks which distinguish the activity before the stimuli (around *t* = 0 s) and during the stimuli, for *t* ∈ (1, 3) s. In α-band, the figure shows more prominent recurrences, such as the patterns around *t* = 1.5 and 2.7 s. We point out that recurrences within δ- and α-bands do not overlap, except in the pre-stimuli period, for *t* ∈ {−0.5, 0} s. Finally, considering all the frequency bands together does not reveal significant similarities of two pre-stimuli. Results from the chi-square test and the *t*-test involving multiple comparison correction are similar. However, it is interesting to note that the *t*-test reveals more significant common recurrences than obtained with the naive chi-square test.

## 4. Discussion

The present work introduces a new recurrence analysis methodology for univariate time series. The first new element is the transformation to a time-frequency representation leading to a multivariate time series of spectral power. This new technique generates a new high-dimensional phase space in which the instantaneous power of the signal evolves. This high-dimensional phase space is mandatory to apply recurrence analysis. In addition, it permits to compute recurrence plots for specific frequency bands. The second new element is the statistical analysis of recurrence plots that takes into account spurious recurrence structures and allows to suppress them. The combination of the two methods permits to extract temporal recurrence structures in data which may reflect underlying transient dynamics in a certain range of frequencies that would have been hidden in conventional methods. To our best knowledge these two techniques have not been considered before.

The first results for two artificial datasets illustrate the methodology and indicate that method detects recurrences in a variable dataset (noise-induced trial variability) by the statistical analysis as seen in Figure 5. These results on artificial datasets prove that the method reveals underlying recurrences in a set of trials if they are present in these trials.

The subsequent analysis of single Local Field Potentials measured experimentally in ferret visual cortex reveals a high trial-to-trial variability, cf. Figure 6. The trial-to-trial variability is surprising due to the well-controlled experiment revealing an intrinsic ongoing activity (Arieli et al., 1995). This result demonstrates that it is mandatory to take into account recurrence variability in several trials. This is done by the methodology proposed. Detailed recurrence analysis of specific frequency bands in Figure 7 reveals missing recurrences in the δ band whereas α-activity exhibits statistically significant temporal recurrence. This important finding reflects a fundamental difference of the nature of δ- and α-activity which has been shown in previous experimental studies on the neural origin of both signal features (Alkire et al., 2000; Ching et al., 2010; Hashemi et al., 2014). Our results suggest that the brain may decode information processing steps in different frequency bands. This might be of importance in previous studies and may shed some new light on neural processes, such as on metastable states in EEG during the emergence from unconsciousness (Hudson et al., 2014) and metastable states in bird songs (Yildiz and Kiebel, 2011).

The effects of pre-stimuli have been hypothesized (Van Rullen et al., 2011; Lundqvist et al., 2013) and we have investigated the effect of pre-stimuli. The performed analysis is based on a rather small set of trials reflecting the responses to identical stimuli. To have sufficiently large dataset for tests, we merged trials coming from two recording sessions. We note that trials coming from two sessions may not be independent, which may introduce errors. We found negligible effects in the δ frequency bands but differences in the temporal recurrence structure in the α frequency band. This result indicates that α-activity is more sensitive to pre-stimuli than δ-activity in the experimental setup under study. This finding is in full line with previous theoretical (Lundqvist et al., 2013) and experimental (Romei et al., 2008) studies on the importance of phase and power of prestimulus α-activity. In addition, we notice the absence of the diagonal line and other strong recurrence patterns visible in Figure 7. This may be the result of merging trials from different sessions, which was necessary to obtain larger test set for the analysis.

The present work shows that trial-to-trial variability in neurophysiological data occurs in spite of well-controlled and simple response-driven experimental conditions and demonstrate how to extract recurrent structures nevertheless. The methodology proposed makes it necessary to choose a well-adapted technique to transform the univariate times series to a multivariate time frequency signal. In addition to our current choice of a spectral reassignment technique, we have employed a conventional wavelet technique using complex Morlet mother wavelets and performed the same recurrence analysis (results are not shown). It turns out that this conventional method does not provide high-quality extraction of transient recurrent structures, given by the reassignment method. This may result from the worse time-frequency resolution of conventional Morlet wavelets. Future work will further investigate the best choice of multi-resolution time-frequency methods. Moreover, the methodology considers surrogate data generated by a temporal random shuffling of data and hence destructing all temporal structure. Future work may include the destruction of the recurrence structure by phase randomization in certain frequency bands (Li et al., 2010).

To conclude, in this work we propose a novel analysis method for trial-to-trial variability of recurrence plots in univariate time series applying a novel statistical analysis technique. This extension of recurrence analysis by a statistical technique is motivated by the fact that many physiological datasets have a limited number of trials but posses the intrinsic recurrence property of patterns of interest. Inspired by the fact that particular physiological patterns very often occur in specific frequency bands, we first build novel recurrence plots from a time-frequency signal representation. A low dimensional time-frequency signal that is built by the band median filter is then used to obtain original trial recurrence plots. Next, we use a chi-squared statistics to obtain statistically important areas of recurrence plots. The work reveals a strong trial-to-trial variability of recurrences in experimental data in spite of the well-controlled experimental paradigm. Moreover, it turns out that recurrences occur in the α-frequency band, whereas activity in the δ-frequency band does not exhibit a temporal recurrent structure indicating frequency-dependent metastable states.

## 5. Data sharing

We provide the time-series of the transient oscillation dataset and Lorenz dataset on the webpage of the corresponding author (https://sites.google.com/site/tamtos/datasets).

## Author contributions

The majority of the analysis steps and the implementations have been performed by TT. KS and FF have provided the experimental data and neurophysiological insights. MF has contributed additional time-frequency analysis results and PB and AH have conceived the study. All authors wrote the manuscript together.

### Conflict of interest statement

The authors declare that the research was conducted in the absence of any commercial or financial relationships that could be construed as a potential conflict of interest.
